# Modulating Brain Connectivity by Simultaneous Dual-Mode Stimulation over Bilateral Primary Motor Cortices in Subacute Stroke Patients

**DOI:** 10.1155/2018/1458061

**Published:** 2018-02-13

**Authors:** Jungsoo Lee, Eunhee Park, Ahee Lee, Won Hyuk Chang, Dae-Shik Kim, Yong-Il Shin, Yun-Hee Kim

**Affiliations:** ^1^Department of Physical and Rehabilitation Medicine, Center for Prevention and Rehabilitation, Heart Vascular Stroke Institute, Samsung Medical Center, Sungkyunkwan University School of Medicine, 81 Irwon-ro, Gangnam-gu, Seoul 06351, Republic of Korea; ^2^Department of Health Sciences and Technology, Department of Medical Device Management & Research, SAIHST, Sungkyunkwan University, 81 Irwon-ro, Gangnam-gu, Seoul 06351, Republic of Korea; ^3^Department of Physical and Rehabilitation Medicine, Kyungpook National University Medical Center, 474 Hakjeongdong, Buk-gu, Daegu 41404, Republic of Korea; ^4^School of Electrical Engineering, Korea Advanced Institute of Science and Technology, Daehak-ro, Yuseong-gu, Daejeon 34141, Republic of Korea; ^5^Department of Rehabilitation Medicine, Pusan National University School of Medicine, Research Institute for Convergence of Biomedical Science and Technology, Pusan National University Yangsan Hospital, Yangsan 50612, Republic of Korea

## Abstract

Repetitive transcranial magnetic stimulation (rTMS) or transcranial direct current stimulation (tDCS) has been used for the modulation of stroke patients' motor function. Recently, more challenging approaches have been studied. In this study, simultaneous stimulation using both rTMS and tDCS (dual-mode stimulation) over bilateral primary motor cortices (M1s) was investigated to compare its modulatory effects with single rTMS stimulation over the ipsilesional M1 in subacute stroke patients. Twenty-four patients participated; 12 participants were assigned to the dual-mode stimulation group while the other 12 participants were assigned to the rTMS-only group. We assessed each patient's motor function using the Fugl-Meyer assessment score and acquired their resting-state fMRI data at two times: prior to stimulation and 2 months after stimulation. Twelve healthy subjects were also recruited as the control group. The interhemispheric connectivity of the contralesional M1, interhemispheric connectivity between bilateral hemispheres, and global efficiency of the motor network noticeably increased in the dual-mode stimulation group compared to the rTMS-only group. Contrary to the dual-mode stimulation group, there was no significant change in the rTMS-only group. These data suggested that simultaneous dual-mode stimulation contributed to the recovery of interhemispheric interaction than rTMS only in subacute stroke patients. This trial is registered with NCT03279640.

## 1. Introduction

Noninvasive brain stimulation (NBS), such as repetitive transcranial magnetic stimulation (rTMS) or transcranial direct current stimulation (tDCS), has recently been adopted for modulating neural excitability in stroke patients [1–3]. After a stroke, interhemispheric imbalance of cerebral cortical excitability occurs and cortical activity in the contralesional hemisphere is abnormally increased [4, 5]. On the other hand, brain activity in the ipsilesional hemisphere is noticeably decreased by interhemispheric inhibition of the contralesional hemisphere [6, 7]. Interhemispheric imbalance of cortical activity induces disruption of interhemispheric connectivity which has been consistently observed in both animals and humans after stroke [8–11].

NBS has been used to recover disrupted interhemispheric balance caused by stroke onset by modulating cortical excitability over specific brain regions. Cortical excitability can be modulated depending on the frequency of rTMS and the tDCS direction of current [12, 13]. This intervention can lead to the improvement of residual motor function by inducing neural plasticity [3, 14–16]. NBS has been mainly performed to restore abnormal interhemispheric balance by facilitating ipsilesional primary motor cortex (M1) excitability or by inhibiting contralesional M1 excitability [14, 17–19]. Recently, dual-site (two sites) or dual-mode (rTMS and tDCS) stimulation has been studied in stroke patients to maximize the brain modulation effect [20–25]. In our previous work, simultaneously applied high-frequency rTMS over the ipsilesional M1 and cathodal tDCS over the contralesional M1 induced better motor recovery compared to high-frequency rTMS only in subacute stroke patients [26].

Changes in cortical networks by NBS over M1 in healthy subjects and stroke patients have been reported in previous studies [2, 5, 27–29]. Even though the results of these studies are diverse depending on participants and protocols, there was a common point indicating that intracortical and interhemispheric connectivity of stroke patients was widely modulated by NBS over the bilateral M1s. Therefore, in this study, we hypothesized that simultaneous stimulation of excitatory rTMS and inhibitory tDCS over bilateral M1 would yield better modulation of interhemispheric balance and interaction in subacute stroke patients compared to the conventional ipsilesional M1 rTMS. This may lead to subsequently better recovery of motor function in subacute stroke patients. We analyzed serial resting-state functional MRI (rs-fMRI) data to define the effects of simultaneous dual-mode stimulation using rTMS and tDCS over bilateral M1 on functional motor network connectivity and efficiency and compare the results to those of conventional rTMS.

## 2. Materials and Methods

### 2.1. Participants

Twenty-four subacute stroke patients were recruited in this study. The inclusion criteria were patients who had suffered their first-ever stroke within 4 weeks of entering the study and had a total Fugl-Meyer assessment (FMA) score [30] under 84. The exclusion criteria were patients who had major active underlying neurological or psychiatric disease, a history of seizure, or metallic implants in their brain. Twelve participants were assigned to the dual-mode stimulation group (10 Hz rTMS over ipsilesional M1 and cathodal tDCS over contralesional M1, 8 males and 4 females, mean age 56.0 ± 13.4 years), and the other twelve participants were assigned to the rTMS-only group (10 Hz rTMS over ipsilesional M1 only, 9 males and 3 females, mean age 54.8 ± 15.5 years) (Table 1). All participants were assessed for the presence of the brain-derived neurotrophic factor (BDNF) Val^66^Met polymorphism via PCR-RFLP using whole blood samples [31]. A previous study demonstrated that this BDNF gene polymorphism negatively influences the effect of rTMS on upper extremity motor recovery in stroke patients [32]. To obtain genetic homogeneity between groups, patients were classified as either valine homozygotes (Val/Val) or methionine allele carriers (Val/Met or Met/Met). There was no significant difference between groups with regard to BDNF genotype frequency (Table 1). Twelve healthy subjects with no history of psychiatric or neurological problems were also recruited as an age-matched healthy control group (8 males, 56.1 ± 14.3 years). The study was performed with the understanding and written consent of all participants, and ethical approval was provided by the Institutional Review Board of Samsung Medical Center.

### 2.2. Experimental Design

A randomized, open-label, parallel study design was adopted in this study. All participants underwent ten daily sessions of stimulation for 2 weeks under one of the two different conditions. In the dual-mode stimulation group, 10 Hz of rTMS was applied over the ipsilesional M1 for 20 minutes with simultaneous application of cathodal tDCS on the contralesional M1. In the rTMS-only group, 10 Hz of rTMS was applied over the ipsilesional M1. We assessed each participant's total FMA score and acquired their resting-state fMRI data at two times: prior to stimulation (prestimulation) and 2 months after stimulation (poststimulation). The FMA scores were assessed under blinded experimental conditions.

### 2.3. Determination of the Location of the Primary Motor Cortex and Resting Motor Thresholds Using Single-Pulse TMS

Each patient was assessed by motor evoked potential (MEP) study using the single-pulse TMS to determine the optimal location of M1 and to evaluate cortical excitability. During stimulation, the patients were seated in a reclining armchair with both hands pronated on a pillow. Electromyography (EMG) data were recorded from the contralateral first dorsal interosseous muscle with surface electrodes. EMG activity was amplified using the Medelec Synergy EMG/EP system (Medelec, Oxford, UK), and the signals were band-pass filtered at 10–2000 kHz. The optimal site (“hot spot”) was determined using a TMS system (Magstim Rapid2 stimulator; Magstim Ltd., Carmarthenshire, UK) and a 70 mm figure-eight coil. The handle of the coil was oriented 45° posterior to the midline because the electromagnetic current flows perpendicular to the central sulcus as described previously [33, 34]. Single-pulse TMS was repeatedly conveyed to the previously determined location to ascertain each patient's resting motor threshold (rMT), defined as the lowest intensity of stimulus necessary to produce a MEP response with a peak-to-peak amplitude of more than 50 *μ*V in five of ten consecutive trials. The examiner monitored muscle activity using real-time EMG. We also evaluated the amplitude and latency of the MEP stimulated with an intensity of 120% of the rMT.

### 2.4. Repetitive Transcranial Magnetic Stimulation

In each session, rTMS was applied to the M1 region of the ipsilesional motor cortex area corresponding to the affected hand, using a Magstim Rapid2 stimulator with two booster modules. Stimulation was delivered at 10 Hz and 90% of the rMT for 5 seconds, with a 55-second intertrain interval. The intensity was kept at a constant 90% of each participant's rMT throughout the trial. For patients in whom an MEP was absent in the ipsilesional hemisphere, the hot spot and rMT were measured using the mirror image of the contralesional hemisphere, as described previously [32]. The hot spot and rMT were assessed prior to initial stimulation and remained identical throughout the trial. A total of 1000 pulses of stimulation were delivered over 20 minutes. This process was repeated 10 times over the course of 10 days in daily sessions. The stimulation was applied to the ipsilesional primary motor cortex (M1) area while researcher was holding the figure-eight coil tangential to the skull. In this study, the rTMS protocols based on safety guidelines for rTMS applications [18].

### 2.5. Transcranial Direct Current Stimulation

The cathodal tDCS was applied to the contralesional M1 using a battery-driven DC stimulator (NeuroConn GmbH, Ilmenau, Germany) that consistently monitored electrical impedance. The anodal tDCS was placed on the supraorbital area over the eyebrow contralateral to the stimulating M1. A constant current flow of 2 mA was delivered for 20 minutes through wet sponge electrodes (size: 7 cm × 5 cm) positioned over the contralesional M1 and the ipsilesional supraorbital area. To reduce discomfort, tDCS stimulation consisted of fade-in and fade-out periods of 5 s.

### 2.6. Resting-State Functional MRI Data Acquisition

Participants were instructed to keep their eyes closed and to remain motionless during the resting-state scan. The resting-state fMRI data were acquired using a Philips ACHIEVA® MRI scanner (Philips Medical Systems, Best, the Netherlands) operating at 3 T. During each session, 100 whole brain images were collected using a T2∗-weighted gradient echo-planar imaging (EPI) sequence: 35 axial slices, slice thickness = 4 mm, no gap, matrix size = 128 × 128, repetition time = 3000 ms, echo time = 35 ms, flip angle = 90°, field of view = 220 × 220 mm^2^. T1-weighted images were also acquired with the following settings: 124 axial slices, slice thickness = 1.6 mm, no gap, matrix size = 512 × 512, field of view = 240 × 240 mm^2^ for atlas transformation.

### 2.7. Data Preprocessing

Preprocessing of resting-state fMRI data was performed using the SPM8 package (Welcome Trust Centre for Neuroimaging, University College London, London, UK). Slice timing correction, spatial realignment for head motion correction, coregistration of the mean image of the fMRI images and a T1-weighted image, spatial normalization into standard template space (resampling to a voxel size of 2 mm isotropic), and spatial smoothing using a 6 mm, full-width half-maximum Gaussian kernel were sequentially performed.

Nuisance signals were removed using linear regression models for nine nuisance parameters. The parameters contained six parameters of rigid body transformation for motion correction, white matter, ventricle, and global signals. Band-pass filtering between 0.009 and 0.08 Hz was performed to obtain synchronized blood oxygen level-dependent signal fluctuations at low frequencies. Nuisance regression and band-pass filtering were processed using Matlab R2014b (The Mathworks, Natick, MA, USA).

## 3. Data Analysis

### 3.1. Construction of the Motor Network

In our study, regions of interest (ROIs) in the motor network were derived from an article described by Rehme et al. [35] that performed meta-analyses on 54 experimental contrasts for movement of the paretic upper limb (472 patients, 452 activation foci) from neuroimaging studies of stroke patients from PubMed search results published up to January 2011. The “affected upper limb movements vs. rest in stroke patients” resulting from the meta-analysis were used in this study. Related regions in the study were not symmetric. Therefore, to obtain network measures from bilateral hemispheres under the same conditions, we constructed a symmetric network by adding the contralesional inferior frontal gyrus (IFG), the inferior frontal sulcus (IFS), the rostral cingulate zone (RCZ), and the ipsilesional anterior intraparietal sulcus (aIPS). The 24 ROIs were defined as 10 mm diameter spheres around the predefined MNI coordinates (Table 2). The lesion area was masked, and network connections were calculated using Pearson's correlation between the mean time courses of each of the 24 ROIs. To determine significant connections, one-sample *t*-tests were performed and *p* value lower than 0.05 was considered significant.

### 3.2. Network Measures

To compare the strength of connectivity between the regions, network values were extracted as follows. Intrahemispheric connectivity of the M1 was measured by average strength of connections between the M1 and predefined ROIs in ipsilateral hemisphere. Interhemispheric connectivity of the M1 was measured by average strength of connections between the M1 and predefined ROIs in contralateral hemisphere. Overall interhemispheric connectivity indicates a mean strength of all connections across the bilateral hemispheres. Interhemispheric connectivity between homotopic regions indicates a mean strength of the interhemispheric connections between homotopic regions of bilateral hemispheres.

The properties of brain networks have been investigated using graph theoretical analysis [36]. This approach is a powerful tool for understanding the reorganization of brain networks during recovery after neurological disorders. Efficiency is a measure of how efficient information is exchanged [37–39]. The efficiency of a network can be defined as follows [38]:

(1) $$\begin{array}{c} E_{global}=\frac{1}{n}\sum_{i\in N}\frac{\sum_{j\in N,j\neq i}{\left(d_{ij}\right)}^{-1}}{n-1}, \end{array}$$

where *n* is the number of regions and *d*_*ij*_ is the shortest path length between region *i* and region *j*. The shortest path length means the average minimum number of connections that must be traveled to move from one region to another [40]. Efficiency was measured using either a weighted motor network or binary motor network. The motor network is originally obtained in the form of a weighted network. A binary network can be created from a weighted network by changing any value greater than zero in the weighted network to one.

### 3.3. Statistical Analysis

The Shapiro-Wilk test was performed to test data for normal distribution. The null hypothesis was rejected in all cases. Repeated measures ANOVA was performed to determine whether there were any significant differences between group (dual-mode stimulation and rTMS-only groups) and time effects (prestimulation and poststimulation) in the resting-state network measures. Paired *t*-tests were used to evaluate within-group differences over time. Post hoc analysis with the Bonferroni correction was also performed. One-way ANOVA was used to compare network measures between groups before stimulation including the healthy control group. The repeated measures ANOVA, paired *t*-tests, and one-way ANOVA were performed using *ranova*, *ttest*, and *anova1* functions, respectively, in the statistics toolbox of Matlab R2014b. The threshold for statistical significance was set at *p* < 0.05 for this study.

## 4. Results

The average FMA scores improved from 43.3 ± 19.5 to 71.8 ± 26.1 in the dual-mode stimulation group and from 42.0 ± 16.9 to 60.0 ± 23.6 in the rTMS-only group. Thus, the dual-mode stimulation group showed a tendency of higher improvement by 11 points in average than the rTMS-only group, even though this difference did not reach to the statistical significance due to lack of participants (*p* = 0.1045).

Before stimulation, there were no differences between patient groups in all network measures of this study. Interhemispheric connections between homotopic regions significantly decreased in both groups compared to the healthy control group. Overall interhemispheric connections and interhemispheric connections of the contralesional M1 decreased in the dual-mode stimulation group compared to the healthy control group.

**Figure 1:**
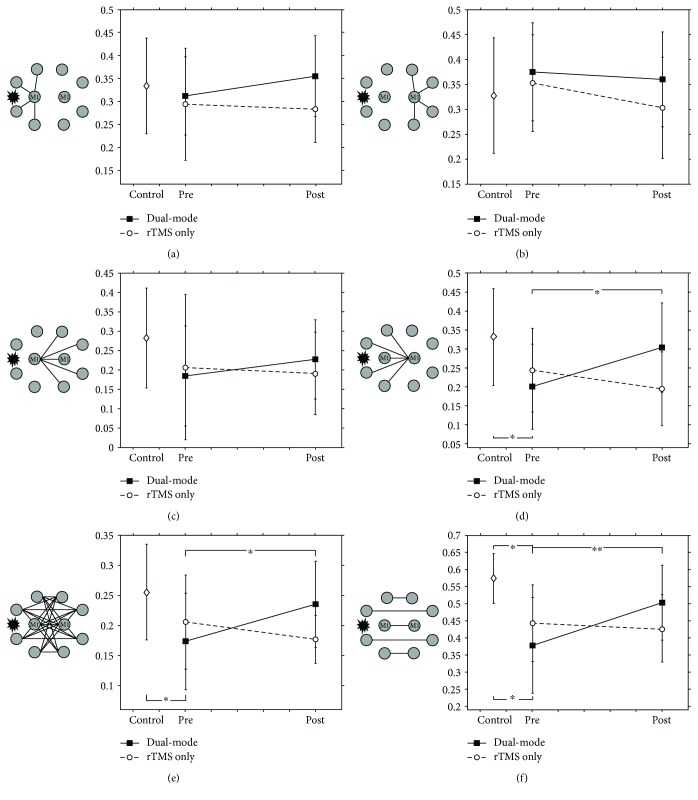
Altered connectivity caused by stimulation. (a) and (b) are the average strength of the intrahemispheric connectivity of bilateral M1. (c) and (d) are the average strength of the interhemispheric connectivity of bilateral M1. (e) and (f) are the average strength of the overall interhemispheric connectivity and interhemispheric connectivity of the homotopic regions. Interhemispheric connectivity of the contralesional M1 and overall interhemispheric connectivity were significantly increased in the dual-mode stimulation group compared to the rTMS-only group poststimulation (^∗^ *p* < 0.05 and ^∗∗^ *p* < 0.01, resp.).

Changes in motor networks after stimulation were investigated in both the dual-mode stimulation and rTMS-only groups. The average strength of intrahemispheric connections for the ipsilesional M1 was slightly increased in the dual-mode stimulation group (Figure 1(a)), and that of the contralesional M1 was slightly decreased in both groups after treatment (Figure 1(b)). However, there were no significant changes in M1 intrahemispheric connectivity in the both groups.

The interhemispheric connectivity of the bilateral M1s was investigated. The average strength of interhemispheric connections of the ipsilesional M1 was slightly increased in the dual-mode stimulation group, but the difference between groups was not statistically significant (Figure 1(c)). On the contrary, the interhemispheric connectivity of the contralesional M1 was drastically increased (*p* = 0.0422) in the dual-mode stimulation group compared to the rTMS-only group (group∗time interactions: *F*_1,22_ = 9.10, *p* = 0.0063, Supplementary Table 1) (Figure 1(d)).

Subsequently, overall interhemispheric connectivity in the motor network and interhemispheric connectivity between homotopic regions that was not restricted to M1 were investigated. To this end, the average strength of overall interhemispheric connections (Figure 1(e)) and interhemispheric connections of homotopic regions in the motor network (Figure 1(f)) was investigated. Interhemispheric connectivity was significantly increased in the dual-mode stimulation group (overall, *p* = 0.0284; homotopic regions, *p* = 0.0074), and the changes showed noticeable increases compared to those of the rTMS-only group (group∗time interactions: overall, *F*_1,22_ = 9.72, *p* = 0.0050; homotopic regions, *F*_1,22_ = 9.57, *p* = 0.0053, Supplementary Table 1).

**Figure 2:**
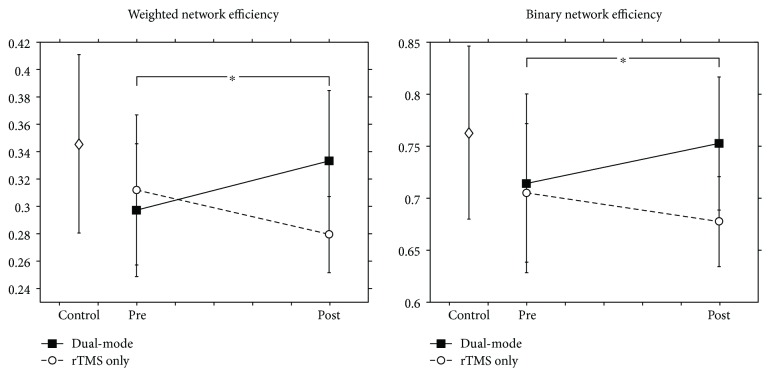
Changes in weighted and binary network efficiencies caused by stimulation. Both weighted and binary network efficiencies were significantly increased in the dual-mode stimulation group compared to the rTMS-only group poststimulation (^∗^ *p* < 0.05).

Network efficiency was measured from prestimulation to poststimulation in both groups (Figure 2). The values of network efficiency in weighted (*p* = 0.0166) and binary (*p* = 0.0284) motor networks were increased after the dual-mode stimulation. Moreover, the increase in the network efficiency in the dual-mode stimulation group was noticeably significant compared to those of the rTMS-only group (group∗time interactions: weighted network efficiency, *F*_1,22_ = 10.91, *p* = 0.0032; binary network efficiency, *F*_1,22_ = 9.80, *p* = 0.0049, Supplementary Table 1).

## 5. Discussion

In conjunction with our previous study which demonstrates the better effect of dual-mode NBS on motor recovery in subacute stroke patients than conventional rTMS [26], this study investigated the alterations in connectivity that occurred in motor networks after NBS. Our results showed that interhemispheric connectivity between the contralesional M1 and the ipsilesional motor-related regions noticeably increased with the additional cathodal tDCS over the contralesional M1 to conventional 10 Hz rTMS on the ipsilesional M1. Overall interhemispheric connectivity and network efficiency significantly increased in the dual-mode stimulation group compared to the rTMS-only group.

Changes in brain connectivity in response to NBS using a single modality (rTMS or tDCS) have been reported in previous neuroimaging studies [41, 42]. NBS induces changes in connectivity between the stimulated region and remote regions. Changes in brain connectivity by NBS over M1 were also investigated [2, 5, 27–29]. Although intracortical activity has consistently showed enhanced activity by M1 facilitation and reduced activity by M1 inhibition, changes in brain networks are diverse depending on participants and protocols. For instance, in stroke patients, tDCS over bilateral M1 increased connectivity of the bilateral sensorimotor networks [28] and interhemispheric connectivity between motor-related regions [29]. Inhibitory rTMS over the contralesional M1 increases connectivity between the ipsilesional M1 and SMA [5]. On the other hand, in healthy subjects, tDCS over bilateral M1 decreased interhemispheric connectivity and inhibitory rTMS decreased intracortical connectivity [27]. Even though these results are diverse, changes of brain networks induced by NBS are considered as biomarkers of motor function changes. After stroke onset, interhemispheric connectivity of the motor network was noticeably disrupted before stimulation compared to the healthy control group. Disruption of interhemispheric connectivity is related to an imbalance of cortical excitability between bilateral hemispheres caused by stroke. These characteristics have been consistently demonstrated in animal and human studies [8–11], and connectivity between hemispheres and cortical regions has also been identified as an important indicator of motor function in stroke patients [8, 9, 43].

In our study, interhemispheric connectivity and network efficiency were significantly recovered by simultaneous dual-mode stimulation of bilateral M1s. This result implies that the dual-mode stimulation effectively helps to rectify interhemispheric imbalance and to change disrupted functional network into efficient network.

In the rTMS-only group, significant alteration of important network measures was not noticed. Recovery of interhemispheric connectivity and improvement of network efficiency differed according to severity of motor impairment in stroke patients [44]. As severity in stroke patients is high, interhemispheric connectivity and network efficiency are difficult to recover. In our study, the severity of stroke patients was relatively high in both groups. Therefore, it may be hard to document the alteration in network measures in this study. However, these network measures were altered in the dual-mode stimulation group. This implies that the simultaneous dual-mode stimulation more effectively modulated the motor network as compared to conventional excitatory rTMS stimulation over the ipsilesional M1. This modulation of the motor network in the dual-mode stimulation group led to better motor recovery compared to the rTMS-only group [26].

Our study had limitation in the experimental design such that there was no sham-control group nor cathodal tDCS-only group. Thus, it is not clear how much the intervention itself made changes in the connectivity of the motor network. Furthermore, only two types of stimulation were compared (dual-mode versus10 Hz rTMS only), which was not sufficient to investigate specific effects of cathodal tDCS only. However, in this experiment, we first targeted to investigate an additional modulation effect of the dual-mode stimulation to the conventional 10 Hz rTMS on brain networks and motor recovery, and the results demonstrated significant differences between the two conditions. Further study for comparisons of multiple conditions can be implicated in the future. This study was also conducted as an open-labeled study but did not apply a double-blind method. This may have affected the participants' states of mind according to different stimulation protocols. However, data was obtained at preintervention and at 2 months postintervention, and the FMA scores were assessed under blinded experimental conditions. Thus, the negative aspects of the open-labeled study were relatively minimized.

## 6. Conclusions

Our results could demonstrate different changes in motor network connectivity induced by NBS in subacute stroke patients. Overall, interhemispheric connectivity and network efficiency, the important indicators of function in the brain networks of stroke patients, were significantly increased in the dual-mode stimulation compared to the rTMS-only group. Therefore, we postulate that simultaneous dual-mode stimulation using both rTMS and tDCS over bilateral M1s may better help subacute stroke patients to overcome interhemispheric imbalance than conventional 10 Hz rTMS over the ipsilesional M1. This evidence may provide insight into multisite or multimode stimulation strategies for enhancing the effects of conventional single-site NBS method in neurorehabilitation of stroke patients.

## Figures and Tables

**Table 1:** Patient characteristics and motor function.

Group	Dual-mode stimulation group	rTMS-only group
Age (years)		
Mean ± SD	56.0 ± 13.4	54.8 ± 15.5
Sex (*n*)		
Male	8	9
Female	4	3
Lesion side (*n*)		
Right	5	7
Left	7	5
Bilateral	0	0
Location of lesion (*n*)		
Cortical	1	2
Subcortical	11	10
Type of stroke (*n*)		
Hemorrhagic	5	1
Ischemic	7	11
Time post stroke (days), mean ± SD		
Prestimulation	20.1 ± 8.7	15.4 ± 5.3
Poststimulation	94.7 ± 10.1	92.3 ± 5.3
Fugl-Meyer assessment scores, mean ± SD		
Prestimulation	43.3 ± 19.5	42.0 ± 16.9
Poststimulation	71.8 ± 26.1	60.0 ± 23.6
BDNF genotype		
Val/Val	4	3
Met allele	8	9

BDNF: brain-derived neurotrophic factor.

**Table 2:** Regions of interest in the motor networks of stroke patients.

Number	Region	Side	MNI coordinates		
			*x*	*y*	*z*
1	Precentral gyrus (M1)	IL	−38	−24	58
2	Precentral gyrus (M1)	CL	42	−14	52
3	Medial superior frontal gyrus (SMA)	IL	−4	−6	54
4	Medial superior frontal gyrus (SMA)	CL	4	−6	54
5	Postcentral gyrus (S1)	IL	−36	−30	60
6	Postcentral gyrus (S1)	CL	40	−28	52
7	Cerebellum (lobule VI)	IL	−24	−60	−22
8	Cerebellum (lobules V and VI)	CL	20	−50	−22
9	Medial superior frontal gyrus (pre-SMA)	IL	−2	6	54
10	Medial superior frontal gyrus (pre-SMA)	CL	2	2	56
11	Dorsolateral precentral gyrus/sulcus (PMd)	IL	−42	−10	58
12	Dorsolateral precentral gyrus/sulcus (PMd)	CL	42	−6	56
13	Ventrolateral precentral gyrus/sulcus (PMv)	IL	−46	−10	48
14	Ventrolateral precentral gyrus/sulcus (PMv)	CL	42	−6	48
15	Parietal operculum (S2)	IL	−48	−18	22
16	Parietal operculum (S2)	CL	50	−28	28
17	Inferior frontal gyrus (IFG)	IL	−48	6	6
18	Inferior frontal gyrus (IFG)	CL	48	6	6
19	Inferior frontal sulcus (IFS)	IL	−50	8	34
20	Inferior frontal sulcus (IFS)	CL	50	8	34
21	Rostral cingulate zone (RCZ)	IL	−8	14	36
22	Rostral cingulate zone (RCZ)	CL	8	14	36
23	Anterior intraparietal sulcus (aIPS)	IL	−42	−40	50
24	Anterior intraparietal sulcus (aIPS)	CL	42	−40	50

IL: ipsilesional side; CL: contralesional side.

## Supplementary Materials

Supplementary Materials Supplementary Table 1: repeated measures ANOVA tables.
