# Supplementary material for: Modulating Brain Connectivity by Simultaneous Dual-Mode Stimulation over Bilateral Primary Motor Cortices in Subacute Stroke Patients
Source: Neural Plast. 2018 Feb 13;2018:1458061. doi: 10.1155/2018/1458061 (PMC5831930; doi:10.1155/2018/1458061)
Supplement: Supplementary Materials — Supplementary Table 1: repeated measures ANOVA tables. [file 1458061.f1.pdf]

Supplementary Table 1. Repeated measures ANOVA tables

Source	Sum of Squares	Degrees of Freedom	Mean Square	F	P
Figure 1A					
Time	0.00322	1	0.00322	0.42	0.5237
Group	0.02424	1	0.02424	2.44	0.1327
Interaction	0.00860	1	0.00860	1.12	0.3012
Error	0.16879	22	0.00767		
Total	0.20485	25			
Figure 1B					
Time	0.01268	1	0.01268	1.74	0.2009
Group	0.01928	1	0.01928	1.62	0.2169
Interaction	0.00354	1	0.00354	0.49	0.4932
Error	0.16046	22	0.00729		
Total	0.19596	25			
Figure 1C					
Time	0.00211	1	0.00211	0.16	0.6895
Group	0.00059	1	0.00059	0.02	0.8758
Interaction	0.01020	1	0.01020	0.79	0.3835
Error	0.28388	22	0.01290		
Total	0.29678	25			
Figure 1D					
Time	0.00892	1	0.00892	1.18	0.2898
Group	0.01250	1	0.01250	0.76	0.3932
Interaction	0.06907	1	0.06907	9.1	**0.0063**
Error	0.16691	22	0.00759		
Total	0.25740	25			
Figure 1E					
Time	0.00342	1	0.00342	1.35	0.2581
Group	0.00211	1	0.00211	0.3	0.5909
Interaction	0.02466	1	0.02466	9.72	**0.0050**
Error	0.05580	22	0.00254		
Total	0.08599	25			
Figure 1F					
Time	0.03575	1	0.03575	5.8	0.0248
Group	0.00029	1	0.00029	0.01	0.9058
Interaction	0.05898	1	0.05898	9.57	**0.0053**
Error	0.13559	22	0.00616		
Total	0.23061	25			
Figure 2 (weighed network efficiency)					
Time	0.00003	1	0.00003	0.02	0.8799
Group	0.00463	1	0.00463	1.49	0.2351
Interaction	0.01417	1	0.01417	10.91	**0.0032**
Error	0.02856	22	0.00130		
Total	0.04739	25			
Figure 2 (binary network efficiency)					
Time	0.00035	1	0.00035	0.26	0.6123

Group	0.02115	1	0.02115	2.79	0.1088
Interaction	0.01311	1	0.01311	9.80	**0.0049**
Error	0.02942	22	0.00134		
Total	0.06403	25			
